# Electrochemical evaluation of proton beam radiation effect on the B16 cell culture

**DOI:** 10.1038/s41598-022-06277-6

**Published:** 2022-02-10

**Authors:** Melania Onea, Mihaela Bacalum, Andreea Luminita Radulescu, Mina Raileanu, Liviu Craciun, Tiberiu Relu Esanu, Teodor Adrian Enache

**Affiliations:** 1 National Institute of Materials Physics, Atomistilor 405A, 077125 Magurele, Romania; 2 Faculty of Physics, University of Bucharest, Atomistilor 405, 077125 Măgurele, Romania; 3 Horia Hulubei National Institute in Physics and Nuclear Engineering, 077125 Măgurele, Romania; 4 Faculty of Biology, University of Bucharest, Splaiul Independentei 91-95, 76201 Bucharest, Romania

**Keywords:** Biochemistry, Biophysics

## Abstract

The interaction of radiation with matter takes place through energy transfer and is accomplished especially by ionized atoms or molecules. The effect of radiation on biological systems involves multiple physical, chemical and biological steps. Direct effects result in a large number of reactive oxygen species (ROS) within and outside and inside of the cells as well, which are responsible for oxidative stress. Indirect effects are defined as alteration of normal biological processes and cellular components (DNA, protein, lipids, etc.) caused by the reactive oxygen species directly induced by radiation. In this work, a classical design of an electrochemical (EC) three-electrodes system was employed for analyzing the effects of proton beam radiation on melanoma B16 cell line. In order to investigate the effect of proton radiation on the B16 cells, the cells were grown on the EC surface and irradiated. After optimization of the experimental set-up and dosimetry, the radiobiological experiments were performed at doses ranging between 0 and 2 Gy and the effect of proton beam irradiation on the cells was evaluated by the means of cyclic voltammetry and measuring the open circuit potential between working and reference electrodes.

## Introduction

Understanding the behavior of biological systems exposed to ionizing radiation requires the development of an appropriate experimental model for the minimization of undesirable effects. Although the biological effects of ionizing radiation on living organisms are known for many years there is a high demand for the development of new devices able to quantify in real-time the effects of radiation on biological systems. The use of high-energy radiation beams to inhibit cancer cell proliferation represents a treatment method that, in some cases, leads to better results than other therapies especially for cancers like hepatocellular carcinoma, non-small cell lung cancer or uveal melanoma^1–5^. Conventional photon (X-ray) radiotherapy comes with increase toxicity to healthy tissue adjacent to the targeted tumor which represents a significant drawback for patients recovering after the treatment^6–9^. Depending on the organs treated by photon radiotherapy, a significant number of organs at risk are identified: lung, heart, bone marrow, etc.^10^. The best approach to decrease the toxicity is to reduce the unwanted irradiation. An alternative more and more used is the use of heavy-particle radiation therapy (most common proton beam therapy) due to its unique performance in tissues^8^. Contrary to photon beam, proton beam is characterized by a finite range in which most of the energy is deposited at the end of the path (generally described as Bragg peak) and without any dose found after exiting the target tissue^8^. The physics of proton beam allows to precisely calculate the depth were all energy will be lost, thus reducing the irradiation of the normal tissue surrounding the tumor, and leading to almost 60% reduction of the internal dose^11^. Despite the accepted advantage offered by the use of proton therapy, there are also a series of disadvantages that need to be considered. Aside from the increase cost to obtain the beam, there are also a number of issues related to the treatment planning of each patient (stopping power, the type of tumor and the surrounding organs, etc.)^12^. Beside these disadvantages, there is a lack of information regarding the mechanisms triggered after proton irradiation in cells. Particularly, production of reactive oxygen species (ROS), known to be caused by any type of ionizing radiation, can be responsible for apoptosis induction, mitochondrial dysfunction, DNA damage, etc.^13, 14^.

Investigating ROS formation in living cells represents a challenging process due to the short life span of the species and their increased reactivity with other molecules or cell components. The main characteristic features of fundamental biological processes are represented by electron-transfer reactions and although the complexity of these reactions varies from case to case, the underlying principles that dictate the rate of electron transfer are the same^15–18^. Consequently, electrochemical techniques can simulate in vitro the redox mechanisms of organisms and provide insight about the electron-transfer reactions^19, 20^. The history of modern voltammetry starts over a century ago with Heyrovsky’s research on the development of polarography^21–26^. Nowadays, due to the simplicity, low-cost, high sensitivity and selectivity voltammetric techniques represent a suitable research approach applied in many scientific fields^27–29^.

The aim of this study was to explore the electrochemical methods as functional analytical tools for the investigation of oxidative stress induced in cell culture exposed to proton radiation and to evaluate the effect of proton beam radiation on the cell cultures. Thus, a classical design of a planar three-electrodes system coupled with a one-compartment electrochemical cell was used to assess human melanoma B16 cells after interaction with hydrogen peroxide and proton irradiation. Considering the studies reported in the literature regarding proton therapy of melanoma cancer cells, B16 cells were chose as a model for this type of cancer. For a better understanding, the voltammetric results were doubled by fluorescence and field emission scanning electron microscopy (FESEM), as well as the conventional fluorescent probes used to detect ROS in cells. In comparison with conventional analysis this approach is promising to produce inexpensive methodologies and can be used for screening purposes.

## Results

In order to underline the potential of electrochemical techniques to be used as a complementary approach for the investigation of reactive oxygen species generated by cells under “extreme” stress conditions, B16 melanoma line (ATTC, Cat, Nr. CRL-6475), a widely used model for human tumors, was chosen for proton irradiation. Thus, the effect of proton beam radiation on the B16 cell cultures was investigated using conventional fluorescent assays and by the means of cyclic voltammetry and open circuit potential evaluation using a planar three-electrode system consisting on a gold working electrode and a platinum-based counter and reference electrodes. Prior to the electrochemical investigation, B16 cells viability at the gold surface was investigated through fluorescence and scanning electron microscopy.

### Cell viability

**Figure 1:**
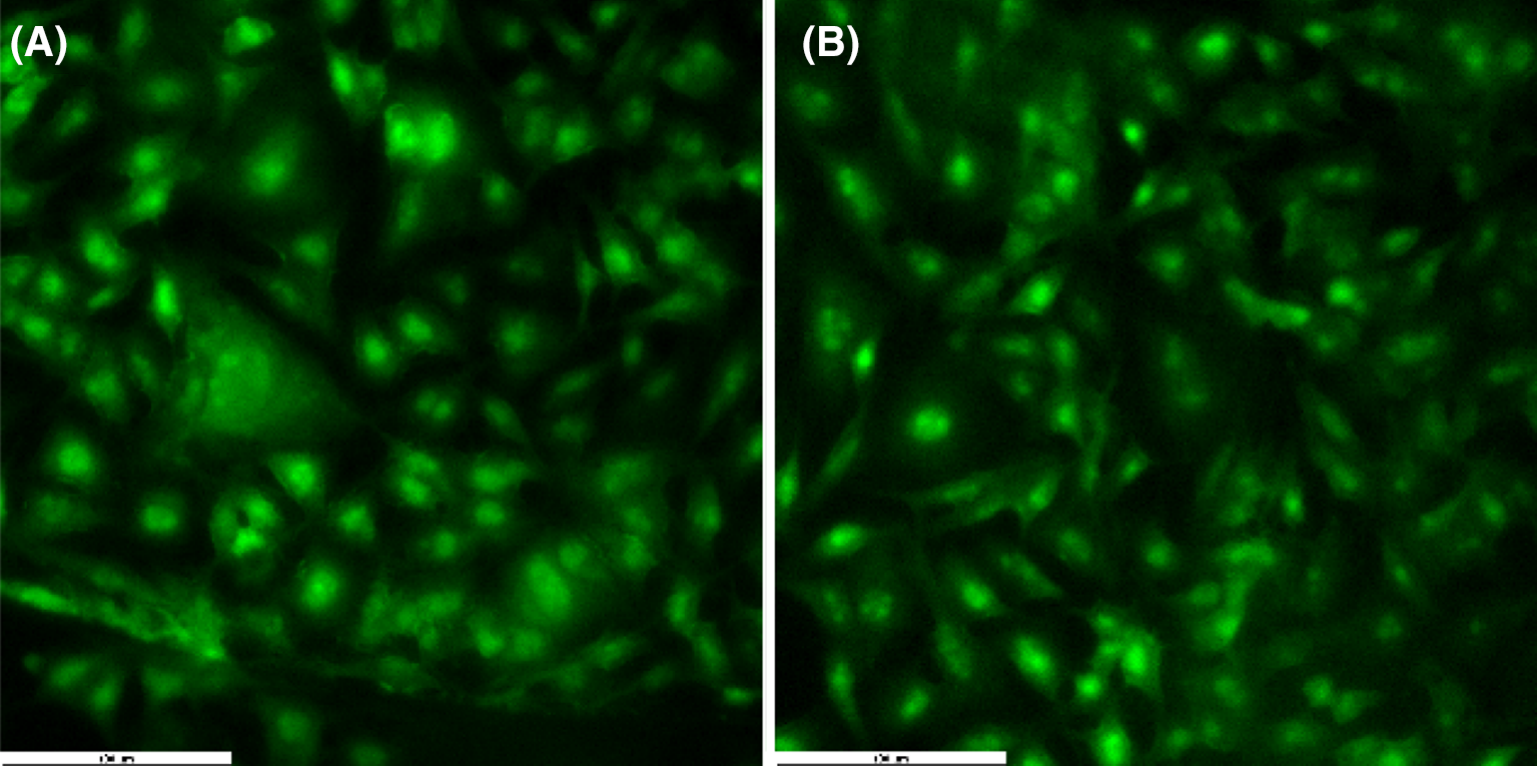
Fluorescence microscopy images of B16 cells grown 48 h on (**A**) biocompatible glass and (**B**) gold surface; scale bars: 100 µm.

The viability of the B16 cells at the gold electrode surface was evaluated and compared with the cells grown on biocompatible glass surface using florescence and scanning electron microscopy. In Fig. 1 are presented the fluorescence images recorded after 48 h for B16 cells grown on the conventional glass slide (Fig. 1A) and on the electrode surface (Fig. 1B). At both surfaces the cells presented the typical morphology, with well-defined nucleus and stellate shaped body with a few dendrites. Comparing the results obtained it was observed that the gold surface showed a high biocompatibility, highlighted by the number of cells and the specific morphology which are similar to the cells grown on the glass slide.

**Figure 2:**
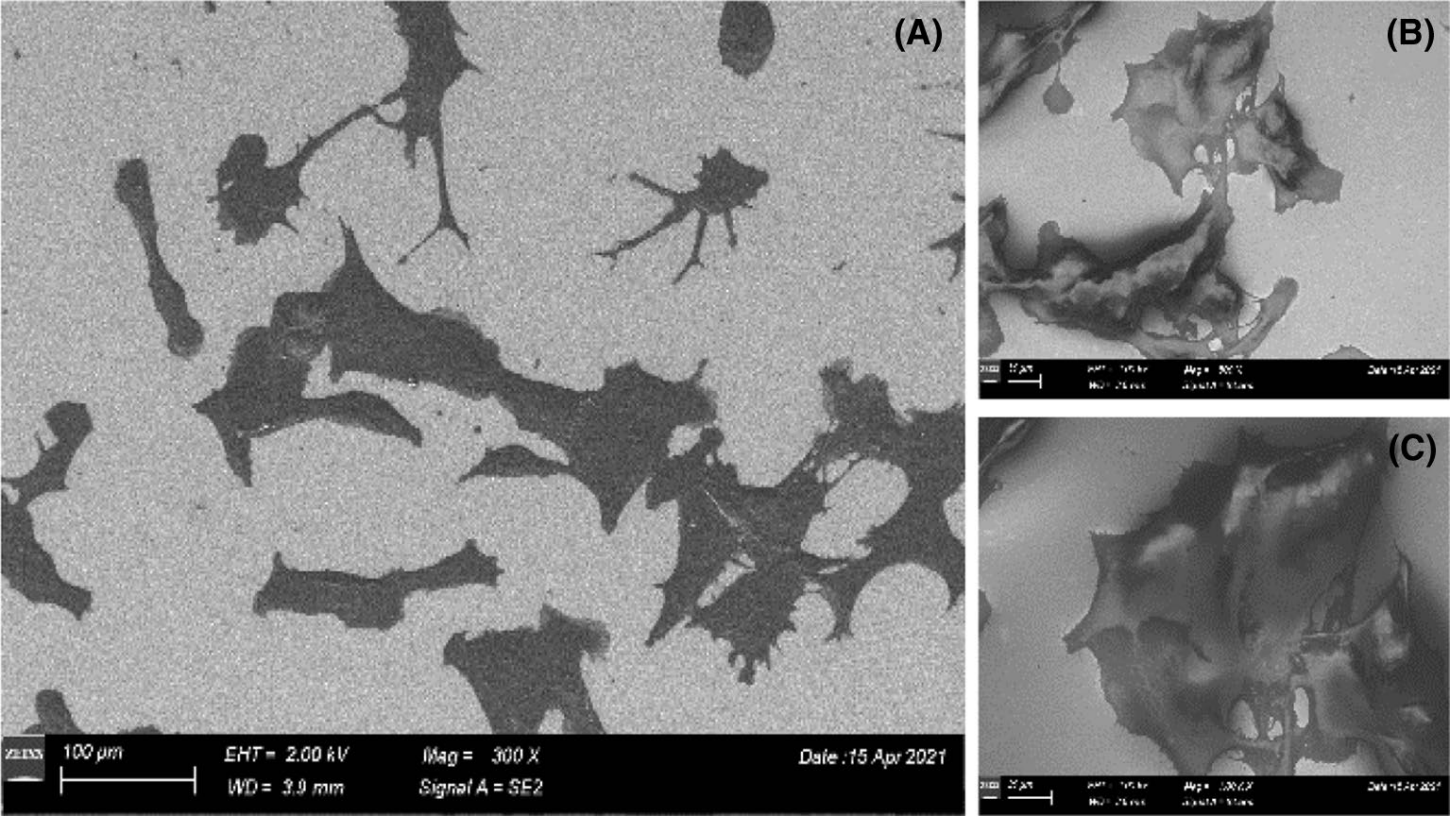
Scanning electron microscopy images of B16 cells grown 24 h on gold surface obtained at different magnifications and using signals from different detectors (SE2 and InLens): (**A**) 300×; (**B**) and (**C**) 500×.

For electron microscopy, the cells were seeded 24 h before Field Emission Scanning Electron Microscopy analysis (FESM), Fig. 2. An advantage of the FESEM system is that it allows the capture of high resolution images at low voltages, thus minimalizing the damage of the biological samples. The secondary electron detector SE2 detects a small component of the back scattered electrons and usually is used to obtain topographic information from the samples, as in Fig. 2A, while the InLens detector is placed on the same axis as the path of the electron beam, and its geometric position allows the detection of the secondary electrons directly form the surface of the sample, as in Fig. 2B,C^30, 31^.

The scanning electron microscopy images revealed that the cells have the expected morphology, a flat body and spread out branches on the surface of the electrode, Fig. 2. These results are in good agreement with the fluorescence images indicating that the gold electrodes are biocompatible and the cells are developing well on their surface.

### Dose response evaluation

The dose response effect of the proton beam radiation on the melanoma B16 cells was investigated using the conventional protocols and assays MitoSOX and H_2_DCFDA.

**Figure 3:**
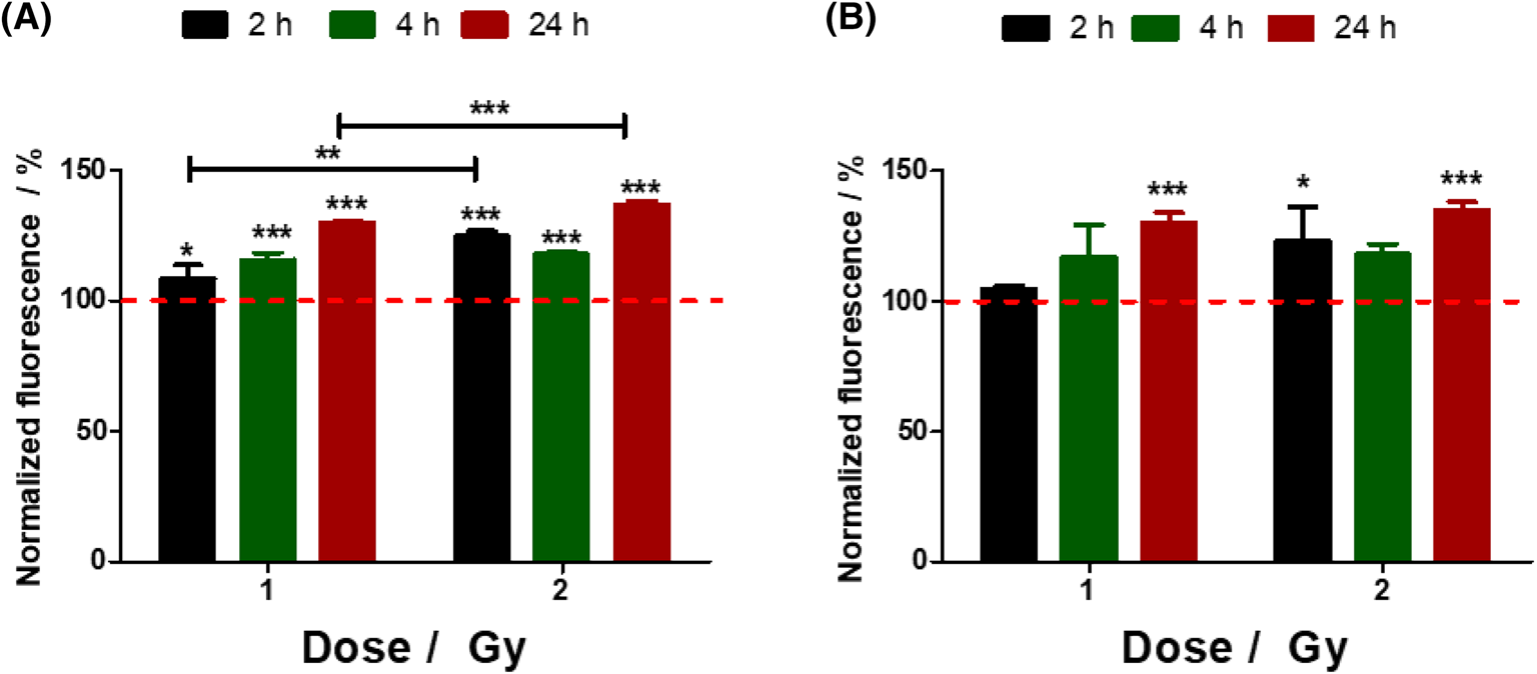
Normalized values to control cells (cells not irradiated) of (**A**) mitochondrial superoxide and (**B**) ROS recorded for B16 cells at 2, 4 and 24 h after proton irradiation at dose rate of 1 Gy/min. Data are reported as mean ± SD. *p < 0.05, **p ≤ 0.01 and ***p ≤ 0.001.

In the MitoSOX assay, a live-cell permeant reagent enters in the cell and selectively target the mitochondria where is oxidized only by superoxide and the reaction product exhibits red fluorescence. After proton irradiation, at a dose of 1 or 2 Gy, the florescence results obtained for mitochondrial stress were recorded at 2 h, 4 h and 24 h. Compared with the control condition (cells not irradiated) it was found that the oxidative stress produced in the mitochondria increases significantly over time and with increasing dose, Fig. 3A.

The total ROS generated by cells upon irradiation was evaluated using H_2_DCFDA protocol, Fig. 3B. The assay was performed using 2′,7′-dichlorodihydrofluorescein diacetate, a nonfluorescent molecule which enters the cells where the intracellular esterases and oxidation cleave the acetate groups releasing the fluorescent molecule 2′,7′-dichlorofluorescein. Similar to MitoSOX, the fluorescence values were normalized to the control cells. Compared with the control condition the highest effects are found for both doses at 24 h after irradiation. The results indicate that upon irradiation the production of reactive oxygen species produced by cells is larger, and will induce more damages.

### Electrochemical evaluation

**Figure 4:**
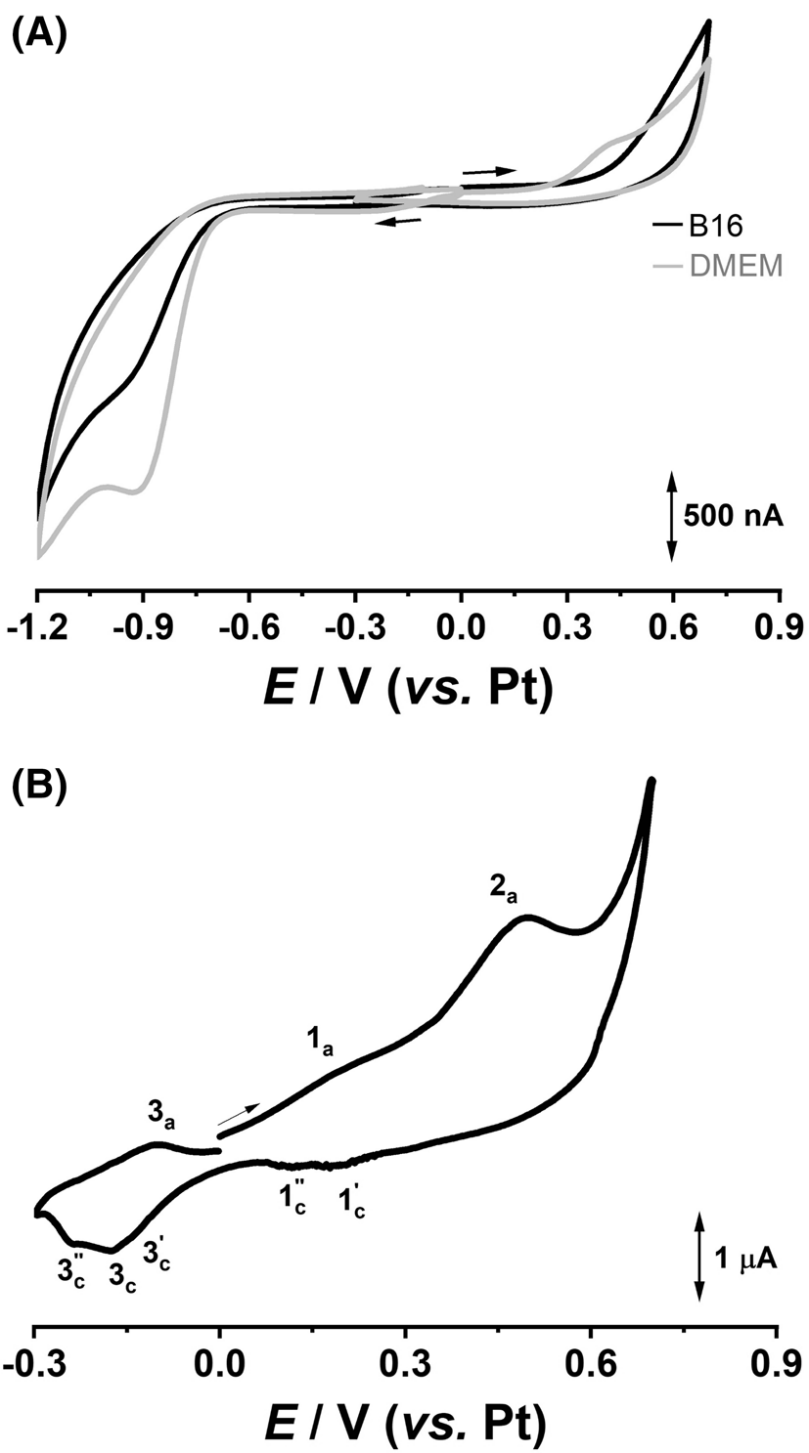
Cyclic voltammograms recorded at gold electrode for (grey line) culture medium and (black line) B16 cells (**A**) before and (**B**) after proton irradiation at a dose of 1 Gy.

Cyclic voltammograms of B16 cells grown for 24 h at gold working electrode surfaces were recorded before and after proton irradiation, Fig. 4, using a planar three-electrode system consisting in a one gold working electrode and a platinum counter and reference electrode. Before each measurement the culture media was removed, cell cultures were washed with PBS and a fresh/new culture medium was added. Nevertheless, the redox behavior of cell culture medium was investigated using the gold working electrode and cyclic voltammetry.

On the positive going scan recorded for cell culture medium between potential limits 0 to + 0.7 V (Fig. 4 A-grey line) one oxidation peak, at E_p_ =  + 0.58 V, corresponding to the electroactive components of the medium^32^, appeared. Reversing the scan direction, from + 0.7 till − 0.3 V no cathodic charge transfer was observed. However, on the negative going scan (Fig. 4 A-grey line) only the oxygen reduction was shown up at E_p_ = − 0.9 V.

The cyclic voltammograms recorded at the same experimental conditions for B16 cells showed the disappearance of the DMEM oxidation peak (Fig. 4A-black line) and a decrease of the oxygen reduction peak current (Fig. 4A-black line) which can be explained by the decrease of the electrode electroactive surface due to the adhesion of the cells.

After proton irradiation the culture medium was removed and a cyclic voltammogram was recorded using a fresh culture medium as supporting electrolyte, Fig. 4B. Scanning toward positive potential values, from 0 till + 0.7 V, two oxidation peaks, at E_p_ =  + 0.20 V and E_p_ =  + 0.50 V were identified. Reversing the scan direction, on the negative-going component of the voltammogram two charge transfer regions, around + 0.15 V and − 0.15 V, containing several reduction peaks, appeared. Continuing the scan, from − 0.3 till 0 V, a new oxidation peak, at E_p_ = − 0.1 V, was observed. Overall, the cyclic voltamogram recorded for B16 cells after proton irradiation reveled at lower potentials two redox pars, 1a/1c and 3a/3c, as well as one oxidation peak 2a occurring at more positive potential, Fig. 4B. Since before irradiation no electron transfer reactions occurred, the charge transfer reactions observed after irradiation correspond to the redox species produced by cells as response to the oxidative stress induced by proton radiation.

Considering the low redox potentials of the reversible pars 1a/1c and 3a/3c these reactions can be associated with the oxidation/reduction of nitro-derivatives and catechol-like species^33, 34^ whereas the anodic reaction, occurring at peak 2a, with the oxidation of hydrogen peroxide^35^.

**Figure 5:**
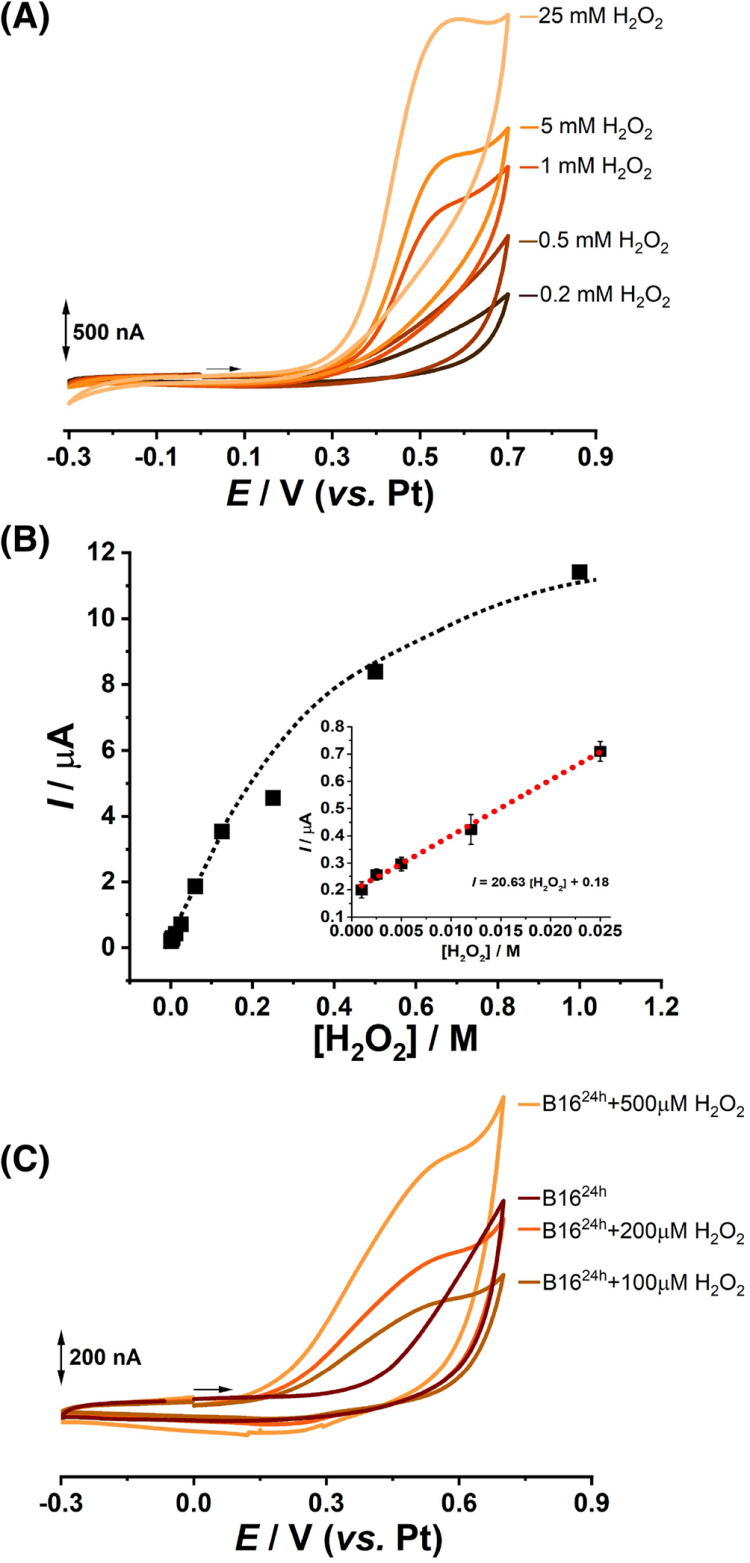
Cyclic voltammograms results recorded at gold working electrodes after 30 min incubation with different concentrations of H_2_O_2_ for (**A**) culture medium with the corresponding (**B**) plot of oxidation current *vs*. H_2_O_2_ concentration and (**C**) B16 cells grown at the electrode surfaces.

The oxidation behavior of hydrogen peroxide at gold electrode was investigated in DMEM cell culture medium at gold working electrode using cyclic voltammetry between potential limits − 0.3 V and + 0.7 V, Fig. 5A. The voltammetric results revealed the fact that the oxidation reaction of H_2_O_2_ takes place in a single step, at *E*_p_ = 0.58 V, and only for concentrations above 1 mM. The data extracted from the five calibration curves, demonstrates, by the values of R^2^ = 0.99, a wider linear range (1–25 mM) following the equation *I*_pa_ = 20.63 [H_2_O_2_] + 0.18 µA, and a limit of detection of 1.82 mM, with relative standard deviation of 8%, was achieved, Fig. 5B.

In order to investigate the effect of hydrogen peroxide on the B16 cells the cells were grown on the gold electrode surface, exposed to H_2_O_2_, and evaluated by the means of cyclic voltammetry. Briefly, after 24 h the cells were washed with PBS, incubated in culture medium with different concentrations of H_2_O_2_, and a cyclic voltammogram was recorded for each electrode after 30 min incubation, Fig. 5C.

The voltammetric results obtained for B16 cells incubated with 100 µM H_2_O_2_ showed a broad oxidation peak occurring at E_p_ = 0.45 V (Fig. 5C-brown line). Increasing the concentration of H_2_O_2_ to 200 and 500 µM (Fig. 5C-dot red line and -orange line) the peak shape become more defined and the oxidation potential shifted to more positive values, 0.5 V and 0.55 V, respectively. On the other hand, on the cyclic voltammogram recorded for culture medium incubated during 30 min with 200 µM H_2_O_2_ (Fig. 5A-black line) no oxidation peaks appeared and only the disappearance of culture medium charge transfer reaction (Fig. 4A-gray line) was observed.

**Figure 6:**
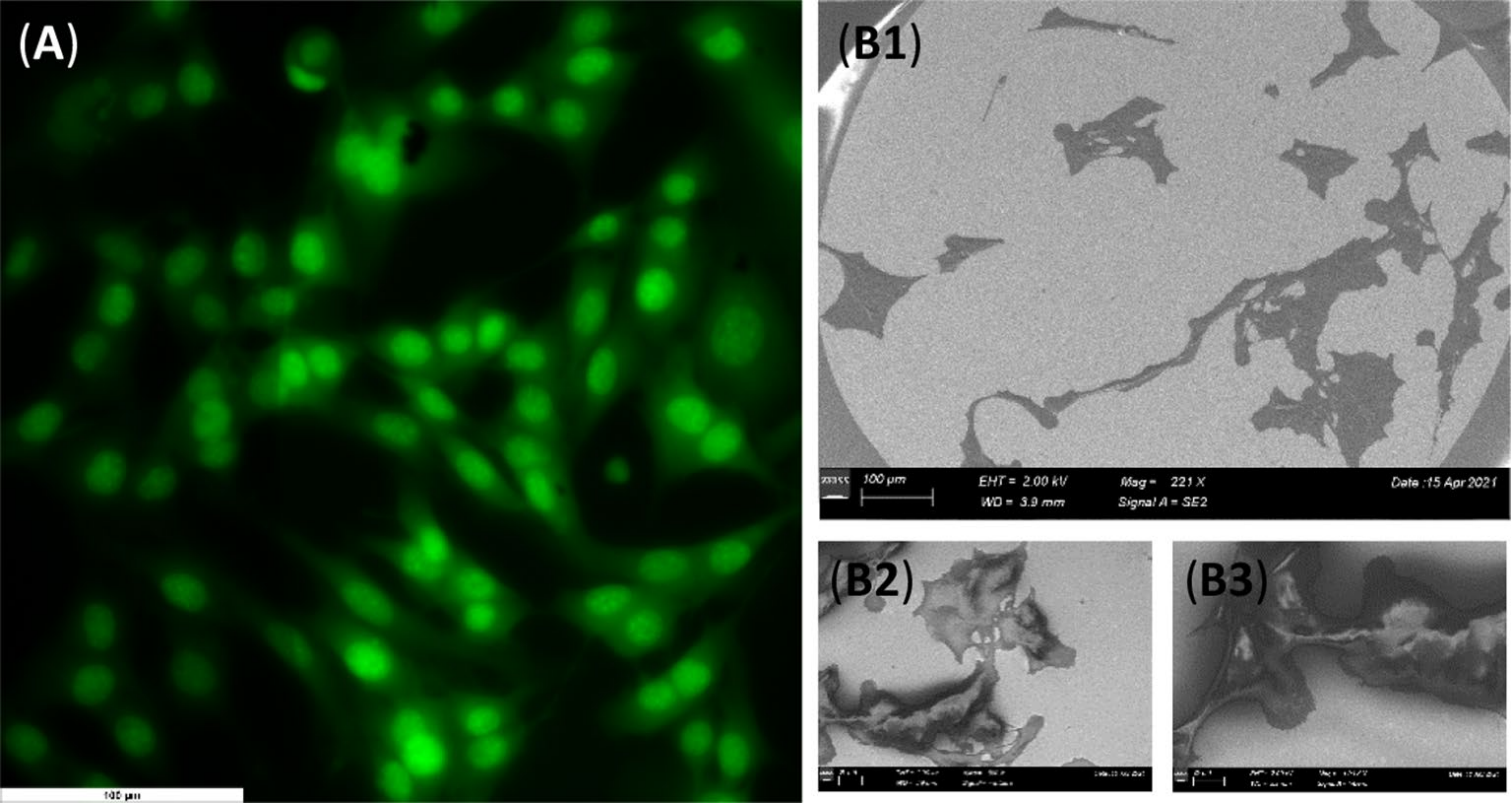
(**A**) Fluorescence microscopy and (**B**) FESEM images obtained for B16 cells at gold surface after interaction with H_2_O_2_; scale bars: 100 µm.

The effect of H_2_O_2_ on the B16 cells grown at gold surface was also investigated through fluorescence and FESEM microscopy. The cells were seeded at a density of 3000 cells and after 48 h for fluorescence and 24 h for FESM the cells were incubated 30 min with 100 µM H_2_O_2_ after which the samples were prepared according to “Electrochemical evaluation” and the microscopy images were recorded, Fig. 6.

The microscopy images revealed the cells having the typical morphology and a well-defined nucleus, Fig. 6. However, comparing with the results obtained before H_2_O_2_ incubation, Figs. 1 and 2, the surfaces presented a lower density of cells, probably due to cellular death induced by oxidative stress.

**Figure 7:**
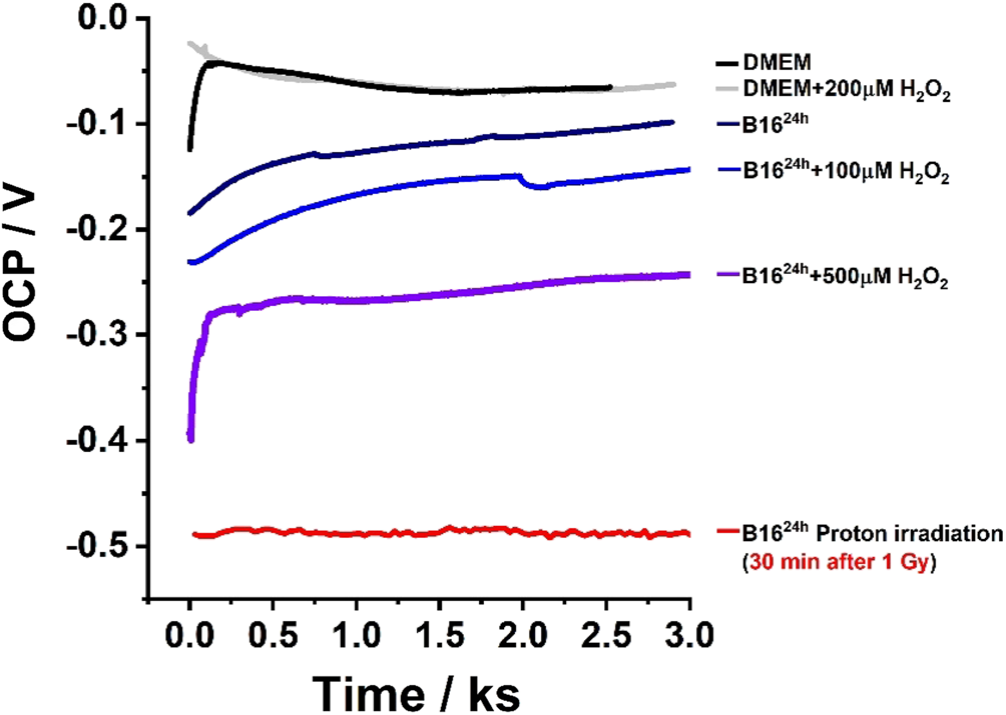
OCP recorded at gold electrodes for culture medium and B16 cells before and after incubation with H_2_O_2_ and proton irradiation (1 Gy at 1 Gy/min).

In a new experiment, the open circuit potential (OCP) between gold working electrode and reference electrode was recorded for B16 cells grown on the working electrode surface, before and after 30 min incubation with H_2_O_2_ and proton irradiation, Fig. 7.

The OCPs recorded for DMEM culture medium before and after addition of 200 µM H_2_O_2_ showed similar values, − 65 mV, (Fig. 7-black line and -grey line) whereas the OCP obtained for B16 cells on the working gold electrode surface presented a more negative potential, − 125 mV at 1.5 ks (Fig. 7-dark blue line). The addition of H_2_O_2_ to B16 cells leaded to a more negative changes of OCP (Fig. 7-blue line) and the highest negative value, − 260 mV, was obtained for 500 µM H_2_O_2_ (Fig. 7-violet line). Similarly, the OCP recorded for B16 cells after proton irradiation (Fig. 7-red line) presented the highest modification of potential value, i.e., − 485 mV, comparing with B16 cells without any treatment.

Since the OCP represents the potential established between the working electrode surface and the environment, the negative shift of the OCP values, appearing with the increase of H_2_O_2_, can be explained taking into consideration the fact that electron exchange occurs between the electrolyte and the surface, resulting in oxidation or reduction of surface compounds. If the overall oxidation current is larger than the reduction current the OCP shifts negatively to produce a zero net current.

## Discussion

**Figure 8:**
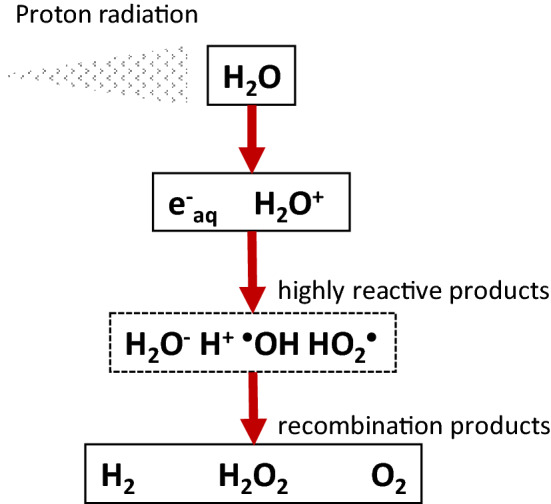
Main reactive species generated by water radiolysis.

The cellular damage induced by ionizing radiation can be caused by the direct or indirect effect of radiation on the cell components, i.e. DNA, protein, lipids. In the direct action, the radiation hits the cell components directly and disrupt their molecular structures. In the indirect action, the radiation hits the water molecules generating free radicals^36^. As ionizing radiation, proton irradiation induces water ionization within and surroundings the living cells. The products of radiolyzed water molecules include the highly reactive, and biologically damaging, water solvated electron (e^−^_aq_), hydroxyl radical (^·^OH), and recombination products such as hydrogen peroxide (H_2_O_2_), Fig. 8.

Moreover, proton radiation represents an alternative therapy to X-rays against different types of cancer, including melanoma. When compared with X-ray, the low-energy proton beam irradiation of melanoma cells resulted in different effects on cell survival, long-term migration or increased elasticity due to actin cytoskeleton rearrangements^37, 38^. On this regard, the effect of oxidative stress on the B16 cell culture induced by hydrogen peroxide or proton beam irradiation was investigated by the means of cyclic voltammetry and through the evaluation of open circuit potential. Firstly, using a classic fluorescent assay, i.e., mitochondrial stress evaluation (see Fig. 3) it was demonstrated that proton irradiation induces reactive species in cells and, as expected, the effect was larger with increasing doses. Following this, the oxidative stress effect was assessed by electro-chemical methods for the B16 cells grown on a gold electrode surface. The electrochemical results were completed and confirmed by fluorescence and field effect scanning electron microscopy.

The results obtained using the cyclic voltammetry and open circuit potential techniques showed a modification of the electrochemical signal after the exposure of the cells to the hydrogen peroxide or proton beam irradiation. For both oxidative stress agonists, i.e., H_2_O_2_ and proton radiation, the cyclic voltammetry showed a main oxidation peak, occurring around + 0.55 V, which may correspond to the oxidation of H_2_O_2_ at the gold surface^39^. However, no oxidation peak was observed on the cyclic voltammogram obtained for 200 µM H_2_O_2_ in culture medium, meaning that the incubation of cells with H_2_O_2_ leaded to H_2_O_2_ production and an increase of extracellular H_2_O_2_ concentration.

Similarly, the measurements of the open circuit potential between the B16 cells grown on gold working electrode and platinum reference revealed a decrease of the OCP values, when compared with control experiments, for B16 cells after incubation with H_2_O_2_ or after proton beam irradiation; the highest negative values being obtained after proton irradiation.

These findings are entirely supported by other studies which demonstrate the fact that exposing the cells to a H_2_O_2_ concentration over 100 µM has a proportionate impact in the release of molecular reactive oxygen species^40–43^. Moreover, since the diffusion rate of hydrogen peroxide through membrane is much faster than the rate of its intracellular consumption, a H_2_O_2_ gradient will be formed across the membrane and the H_2_O_2_ concentration will be equal extracellularly and intracellularly.

Generally, the electrochemical sensing of hydrogen peroxide, as enzymatic coproduct^44, 45^ or released by biological cells as response to stress^46–48^, is achieved by amperometric measurements of the reduction current of H_2_O_2_ and involve the use of different nanomaterials like metal nanoparticles, carbon nanotubes, graphene, etc. Depending on the reduction potential of H_2_O_2_, the removal of the oxygen from the electrochemical cell may be necessary. However, the metal-based electrodes used in this work are fabricated on a biocompatible glass substrate by thin-film deposition technologies capable to produce high quality nanostructured gold electrodes with high performance towards anodic detection of H_2_O_2_, without oxygen interferences.

## Conclusions

Electrochemical techniques, especially voltammetry and the related techniques have appropriate features to be used as monitoring methods for biological systems where the investigation of the short life span chemical species, generated through molecular interactions, represents a challenging process.

In this study it was showed the efficiency of using cyclic voltammetry and/or OCP to investigate the generation of ROS induced by H_2_O_2_ or proton radiation in B16 cell culture. All the measurements were recorded at room temperature and atmospheric CO_2_ partial pressure. The results are encouraging and the electrochemical recording system needs to be improved to allow, in the future, real time observation of the cell cultures for a longer time span under controlled conditions such as relative humidity, 5% CO_2_ and 37 °C. The development of a such system is promising to produce an inexpensive biotechnological device to be used in quality monitoring of cell cultures by combining the specificity of biological cells with the powerful properties of electrochemical techniques and will lead to the develop of different biosensors, as functional analytical tools, for quality control of cell cultures.

## Materials and methods

### Materials

For in vitro studies we used B16 melanoma cell line (cat. Nr. CRL-6475), ATCC (USA). Other materials used were: Dulbecco’s Modified Eagle Medium (DMEM) from Sigma-Aldrich, phosphate buffer saline (PBS) from Thermo Fisher Scientific, fetal bovine serum (FBS) from Thermo Fisher Scientific, trypsin (cat. Nr. BE17-160E) from Lonza Bioscience Solutions, antibiotics from Biological Industries, and formaldehyde (catalog Nr. 252549), glutaraldehyde (cat. Nr. 340855) from Sigma-Aldrich and osmium tetroxide (cat. Nr. 251755) from Merck (Sigma-Aldrich). For all solutions, when necessary, sterile purified water from a Milli-Q system (conductivity less than 0.1 μS cm^−1^) was used. The planar three electrode system (ED-SE1-AuPt) consisting in one gold working electrode and two platinum (counter and reference) electrodes was acquired from MicruX Technologies, Spain.

### Instrumentation

#### Voltammetric parameters and electrochemical cells

The measurements were performed using a computer controlled Ivium potentiostat with IviumSoft version 2.219 (Ivium Technologies, Eindhoven, The Netherlands). Measurements were carried out using the planar electrodes (ED-SE1-AuPt) and the electrochemical interfaces platform (ED-AIO-CELL-1x), Micrux, Spain. Before each experiment, the electrodes were sterilized with 70% ethanol/water, for several hours, and rinsed with sterile Milli-Q water.

Cyclic voltammograms were recorded using a scan rate of 100 mV s^−1^ with a 2 mV step potential and the open circuit potential (OCP) was measured between gold working electrode and platinum reference electrode. Except Fig. 4B, all voltammograms were recorded 3–5 times and presented as average. The relative standard deviation was between 1 and 15%.

#### Fluorescence microscopy

The fluorescence microscopy images were obtained using a Leica DM6B upright fluorescence microscope (Leica Microsystems CMS GmbH) equipped with a Leica CTR6 LED (electronic box containing the power supply for the electronics and the lamps) and a Leica EL6000 external light source for fluorescence excitation. The samples were imaged using a 40× objective (0.65 NA, 0.36 mm WD and correction ring) from Leica, the appropriate filter cube (excitation filter 480/50 nm, dichroic mirror 505–510 nm and emission filter 527/30 nm) and the 4.2 MP sCMOS Leica DFC9000 monochrome fluorescence camera.

#### Field emission scanning electron microscopy (FESEM)

The morphology of the samples was investigated using a Gemini 500 Carl Zeiss Field Emission Scanning Electron Microscope (FESEM) working in both High Vacuum (HV) and Variable Pressure (VP) modes, from 0.2 to 30 kV, equipped with LaB6 filament, NanoVP mode, InLens and SE2 detectors.

#### Fluorescence spectrometry

MitoSOX and ROS measurements were performed using a Mithras LB 940 plate reader (Berthold) equipped with the appropriate filters to record the probe fluorescence.

#### Experimental setup for cell proton irradiation

The cells were irradiated using a TR19 cyclotron from Radiopharmaceuticals Research Center, IFIN-HH. The cyclotron is equipped with a 6 m horizontally external beam line for interdisciplinary research that transfer the protons from the cyclotron vault in a separate bunker.

The extension for biological irradiation is equipped with a full automatic linear axes of motion that allow the precise positioning of the wells and an Advanced Markus ionization chamber used to calibrate the proton beam. The cells were irradiated with a dose rate of protons of 1 Gy/min that is equivalent with a current of 11.2 pA. The proton energy extracted in air, before the cell culture plate was 16.2 MeV.

### Procedures

#### Cells cultivation and proton irradiation

The B16 melanoma cells were grown in Dulbecco's Modified Eagle Medium (DMEM) culture medium supplemented with 4.5 g/L glucose, 2 mM l-glutamine, 10% fetal bovine serum and penicillin (100 U/mL), streptomycin (100 μg/mL), under controlled conditions (humidity, 5% CO_2_, 37 °C). Sub-cultivation was done in cell culture flasks (T-25) and when cells reached pre-confluence ~ 80% the cells were detached using trypsin solution of 0.25% concentration, counted and plated for in plates or one the electrodes for the experimental procedures. For in vitro studies of ROS, the cells were plated in 12 well plates at a density of 40,000 cells/ well and placed in the incubator at 37 °C for 24 h prior to irradiation.

For the electrochemical and microscopy experiments, B16 cells were seeded on the electrode surface (d = 1 mm) at a density of 3000 cells/cm^2^ and allowed to adhere at the surface. After 10 min culture medium was added to cover the entire electrode and the cells were placed in the incubator at 37 °C for 24 or 48 h, respectively, under controlled humidity and 5% CO_2_. For microscopy experiments, B16 cells were also seeded on a conventional biocompatible glass slide in order to investigate the biocompatibility of the electrodes.

**Figure 9:**
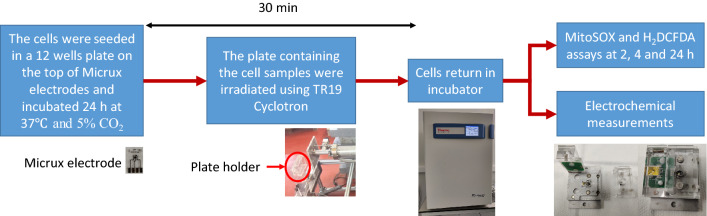
The workflow chart of the experimental steps.

The cells grown in the 12 well plate were irradiated with doses of 1 and 2 Gy, at a dose rate of 1 Gy/min, while the cells grown on the electrode were irradiated with a dose of 1 Gy at a dose rate of 1 Gy/min. A workflow chart presenting the experimental steps is provided in Fig. 9.

#### Cells fixation for fluorescence microscopy

After 48 h of cell growth on the electrode surface, electrodes were washed in a phosphate buffer saline solution. Fixation of the grown cells was performed by incubating the electrodes at room temperature for 15 min using a solution of 3% paraformaldehyde 0.2% glutaraldehyde in PBS. Then the cells were washed with PBS and stained with a solution of 2 mg/mL Acridine-Orange, in the dark for the 20 min. Finally, the cells were washed with PBS and mounted on a glass slide and imaged at the microscope.

#### Cells fixation for FESEM

For FESEM assay the B16 cells were seeded on the gold working electrode following the cell cultivation protocol mentioned above, washed with PBS after 24 h incubation and fixed for 10 min with 3% formaldehyde and 0.2% glutaraldehyde. In order to avoid the polarization and the damage of the sample under electron beam, a 20 min post-fixation, using 0.1% OsO_4_, was necessary. The OsO_4_ treatment was preferred to any metal coating that could interfere with the surface of the cells.

#### Measurement of mitochondrial superoxide and ROS

After irradiation, the production of superoxide molecules in mitochondria was investigated at three different time points (2 h, 4 h and 24 h). Briefly, the medium was removed and over the cells was added a 5 µM solution of MitoSOX (MitoSOX™ Red reagent, Invitrogen™), and incubated for 30 min at 37 °C. After the incubation period, the solution was removed and the cells were lysed with trypsin. Finally, the fluorescence of the samples was measured in a plate reader (Mithras LB 940, Berthold) using the following conditions: λ_ex_ = 485 nm/λ_em_ = 590 nm.

At similar time points we also investigated intracellular H_2_O_2_ and oxidative stress using H2DCFDA (Invitrogen™). Briefly, cells were incubated 30 min with 50 µM of the reagent in a humidified incubator under an atmosphere containing 5% CO_2_ and protected from light, then the cells were trypsinized and the fluorescence was measured in a plate reader (Mithras LB 940) using the following conditions: λ_ex_ = 485 nm/λ_em_ = 535 nm.

### Statistical analysis

Data are presented as the mean ± standard deviation (SD). Statistical analysis was performed using GraphPad Prism 5 software (GraphPad Software, USA). The statistical significance of differences between experimental groups was calculated using One-way analysis of variance with Tukey's Multiple Comparison Test. The values of p < 0.05 were considered statistically significant.

## Data Availability

The data presented in this study are available on request from the corresponding author.
