# Author Correction: Electrochemical evaluation of proton beam radiation effect on the B16 cell culture

**DOI:** 10.1038/s41598-022-14652-6

**Published:** 2022-06-14

**Authors:** Melania Onea, Mihaela Bacalum, Andreea Luminita Radulescu, Mina Raileanu, Liviu Craciun, Tiberiu Relu Esanu, Teodor Adrian Enache

**Affiliations:** 1 National Institute of Materials Physics, Atomistilor 405A, 077125 Magurele, Romania; 2 Faculty of Physics, University of Bucharest, Atomistilor 405, 077125 Măgurele, Romania; 3 Horia Hulubei National Institute in Physics and Nuclear Engineering, 077125 Măgurele, Romania; 4 Faculty of Biology, University of Bucharest, Splaiul Independentei 91-95, 76201 Bucharest, Romania

Correction to: *Scientific Reports* https://doi.org/10.1038/s41598-022-06277-6, published online 10 February 2022

The Acknowledgments section in the original version of this Article was incomplete.

“Financial support from the Operational Programme Competitiveness 2014–2020, Project NANO-BIOSURF-SMIS 103528 and Executive Agency for Higher Education and Research Funding (UEFISCDI) and National Research Council (CNCS) the Core Program 2019–2022 (contract 21N/2019) and the project PN-III-P4-ID-PCE-2020-1403, within PNCDI III, are gratefully acknowledged.”

now reads:

“Financial support from the Operational Programme Competitiveness 2014–2020, Project NANO-BIOSURF-SMIS 103528 and Executive Agency for Higher Education and Research Funding (UEFISCDI) and National Research Council (CNCS) the Core Program 2019–2022 (contract 21N/2019) and the project PN-III-P4-ID-PCE-2020-1403, within PNCDI III, are gratefully acknowledged. The fee for open access publication was supported from the project 35PFE/2021, funded by the Romanian Ministry of Research, Innovation and Digitization.”

The original Article has been corrected.

